# Prognosis and Phenotypes of Advanced Head and Neck Carcinoma Associated With Hypercalcemia

**DOI:** 10.1002/hed.28126

**Published:** 2025-03-11

**Authors:** Elodie Mamou, Paul Gougis, Baptiste Abbar, Jean‐philippe Spano, Laetitia Morardet, Aurore Vozy

**Affiliations:** ^1^ Department of Medical Oncology, Sorbonne Université, Assistance Publique‐Hôpitaux de Paris (AP‐HP), Pitié Salpêtrière Hospital Paris France; ^2^ Department of Pharmacology, Sorbonne Université, Institut National de la santé et de la Recherche médicale (INSERM), Assistance Publique–Hôpitaux de Paris (AP‐HP), Clinical Investigation Center (CIC‐1901), Pitié‐Salpêtrière Hospital Paris France; ^3^ Residual Tumor & Response to Treatment Laboratory, RT2Lab, INSERM, U932 Immunity and Cancer, Institut Curie, Université Paris Cité Paris France; ^4^ Centre d'Immunologie et Des Maladies Infectieuses (CIMI‐Paris), Sorbonne Université Paris France

**Keywords:** hypercalcemia: Head and neck squamous cell cancer, overall survival, retrospective study

## Abstract

**Purpose:**

Hypercalcemia is the most common metabolic disorder in cancer, affecting 10%–20% of patients with advanced malignancies, including squamous cell carcinoma of the head and neck (HNSCC), though its prognostic significance remains poorly studied. This study aimed to evaluate the prognostic impact of hypercalcemia at diagnosis in patients with locally advanced or metastatic HNSCC and to explore underlying mechanisms and treatment options.

**Methods:**

We conducted a bicentric, retrospective analysis of patients diagnosed between 2015 and 2021, including those with locally advanced or metastatic HNSCC undergoing chemotherapy. Hypercalcemia at diagnosis was defined as an albumin‐corrected serum calcium level > 2.6 mmol/L, equivalent to 10.4 mg/dL. The primary outcome was overall survival (OS), compared between hypercalcemic and non‐hypercalcemic patients using multivariate analysis. Progression‐free survival (PFS), along with clinical, biological, and therapeutic characteristics were also evaluated.

**Results:**

The study included 286 HNSCC patients, 225 (78.7%) of whom were male. Hypercalcemia incidence was 17.8%. The median OS for the cohort was 7.9 months (95% CI = 6.8–10.6). Hypercalcemic patients had a median OS of 5.9 months (95% CI = 3.8–7.9), compared to 9.1 months (95% CI = 7.3–11.7) in non‐hypercalcemic patients (*p* = 0.002). In multivariate analysis, hypercalcemia was associated with worse OS (HR = 1.73, 95% CI = 1.17–2.56, *p* = 0.006). Median PFS was 4.3 months (95% CI = 3.6–5.5) for all patients. Hypercalcemic patients had a significantly shorter PFS of 2.4 months (95% CI = 1.9–4.8) compared to 4.7 months (95% CI = 3.8–5.9) in non‐hypercalcemic patients (*p* = 0.0025). Multivariate analysis identified hypercalcemia and oral cavity tumors as negative prognostic factors, with HRs of 1.76 and 1.86, respectively. Bone metastasis rates were similar (17.6% vs. 16.2%), but local osteolysis was significantly higher in hypercalcemic patients (54.1% vs. 27.2%, *p* = 0.003). Bisphosphonates were administered to 38% of hypercalcemic patients.

**Conclusion:**

In this study, hypercalcemia was an independent negative prognostic factor of survival and rapid progression in patients with locally advanced or metastatic HNSCC.

## 1 Introduction

Hypercalcemia is the most common life‐threatening metabolic disorder observed in cancer patients, affecting approximately 10%–20% of individuals with advanced‐stage malignancies [1]. While the incidence of hypercalcemia is relatively low at early stages of cancer (1%–5%), it increases significantly in patients with advanced disease, reaching rates of up to 30%–50% [1]. Hypercalcemia in cancer patients mainly results from increased bone resorption, where calcium release from the bone exceeds the kidneys' excretory capacity [2]. Four mechanisms are responsible: Local osteolysis, humoral hypercalcemia driven by parathyroid hormone‐related protein (PTH‐rP), hypercalcemia mediated by 1.25‐dihydroxyvitamin D, and ectopic hyperparathyroidism, though the latter two are rare [3, 4]. Local osteolysis occurs when malignant cells disrupt calcium regulation through osteoclastic bone resorption, further promoted by tumor cells interacting with bone cells and cytokine secretion [5]. PTH‐rP secretion by tumors mimics parathyroid hormone action, increasing bone resorption and calcium reabsorption in the kidneys [6]. It presents a wide range of clinical symptoms, including abdominal pain, vomiting, polyuria, and polydipsia. However, more severe manifestations can occur, such as progressive mental impairment, renal failure, and, in extreme cases, coma or ventricular fibrillation leading to cardiac arrest [7]. The management of hypercalcemia typically involves aggressive rehydration and the administration of bone resorption inhibitors, primarily bisphosphonates [8].

Head and neck squamous cell carcinoma (HNSCC) is the sixth most common cancer globally and ranks as the seventh leading cause of cancer‐related mortality [9]. Hypercalcemia is a well‐recognized complication in HNSCC, with reported incidences ranging from 3% to 50% [10, 11]. Hypercalcemia in patients with HNSCC is often associated with poor prognosis, with a median survival of 2–6 months following diagnosis [9, 12]. Despite its frequency, the characteristics and clinical outcomes of hypercalcemic patients with HNSCC remain poorly described, and there is a need to investigate strategies to improve both life expectancy and management of hypercalcemia‐related complications in this patient population.

This study aims to evaluate the prognostic impact of hypercalcemia at diagnosis in patients with recurrent or metastatic HNSCC. Additionally, we seek to explore the clinical characteristics, underlying etiologies, and treatment approaches associated with this condition.

## 2 Patients and Methods

### 2.1 Study Design and Population

This retrospective study included all consecutive patients diagnosed with head and neck squamous cell carcinoma (HNSCC) and treated between 2015 and 2021 in the Medical Oncology Departments of Pitié‐Salpêtrière and Tenon Hospitals, both part of Assistance publique—Hôpitaux de Paris. The inclusion criteria were histologically confirmed HNSCC, age ≥ 18 years, and recurrent or metastatic disease. Patients were excluded if they had tumor localizations outside the oral cavity, larynx, oropharynx, or hypopharynx, or if they presented with different histologies (mixed cases were excluded). Other exclusion criteria were patients who received curative‐intent treatments (e.g., surgery or radiotherapy), those who did not undergo first‐line systemic treatment and were only managed with palliative care, patients not primarily followed at the two participating centers, and patients with other concurrent active cancers requiring treatment. Hypercalcemia was defined as albumin‐corrected serum calcium levels over 2.6 mmol/L, equivalent to 10.4 mg/dL. Relevant clinical, epidemiological, and therapeutic data were retrospectively collected. Data extracted from patient medical records included the biological sex and age category of patients, ECOG (Eastern Cooperative Oncology Group), tumor site, metastatic status, presence of bone metastasis, osteolysis at diagnosis, and calcemia. The Combined Positive Score status (CPS), employed as a predictive score for response to immunotherapy, was collected. The calcemia category classified these levels into normal or abnormal ranges. Regarding treatment, the first line of treatment, treatment discontinuation, number of treatment lines, and treatment dose reduction were recorded.

### 2.2 Endpoints

The primary endpoint of the study was to evaluate overall survival (OS) in patients with or without hypercalcemia at the time of diagnosis of recurrent or metastatic disease evaluated in a multivariate analysis. The secondary endpoint was to assess progression‐free survival (PFS) following first‐line systemic therapy. Additionally, clinical and therapeutic factors associated with the occurrence of hypercalcemia in these patients were collected and analyzed.

### 2.3 Statistical Analysis

Overall survival (OS) and progression‐free survival (PFS) were estimated using the Kaplan–Meier method, with comparisons between survival curves performed using the log‐rank test. Multivariate Cox proportional hazard models were employed to estimate hazard ratios (HRs) with 95% confidence intervals (CI). Variables explored within the multivariate analysis were the following variables at tumor diagnosis: Sex (biological, at birth), age, ECOG performance status (0–1 vs. 2+), tumor site, presence of metastasis, bone metastasis, osteolysis, the first line of treatment, platinum‐refractory, and initial dose adaptation. Association between hypercalcemia and various clinical variables were assessed using Fisher's exact test for categorical variables and the Mann–Whitney U test for continuous variables. All statistical analyses were conducted using R software (version 4.3.1) with the “survminer” package.

### 2.4 Ethics Statement

All living patients were provided with written information and gave verbal consent for data collection. Patients' clinical records were retrospectively gathered from electronic files using de‐identified forms. This study complies with French regulations under the MR004 framework, for which formal ethical approval is not required. The study database was declared to the “Commission Nationale de l'Informatique et des Libertés” (CNIL No. CERFA 13810*01).

## 3 Results

### 3.1 Patient Characteristics

**FIGURE 1:**
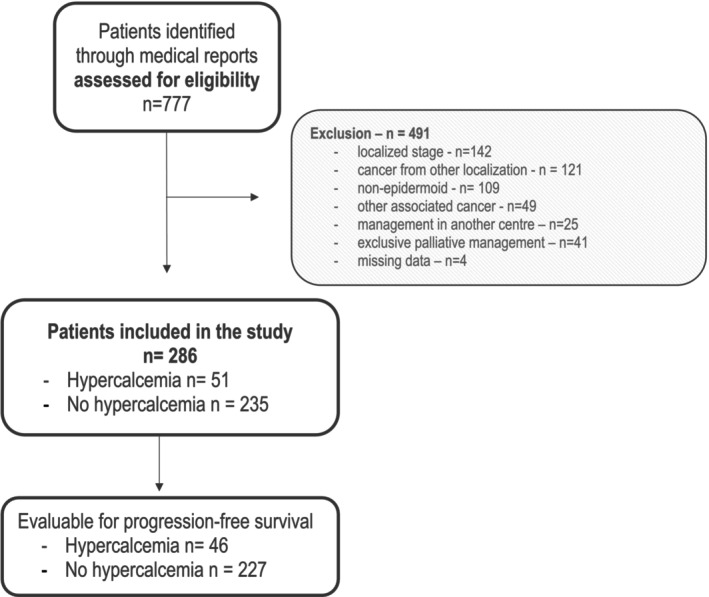
Flow chart.

From January 2015 to December 2021, a total of 777 patients were identified through the local database. Of these, 372 patients met the inclusion criteria, while 119 were excluded due to the presence of another concurrent cancer, management at a different center, exclusive palliative care, or incomplete data (as detailed in Flow Chart Figure 1). Ultimately, 286 patients were included in the study. The median follow‐up period was 7.9 months (95% CI = 6.8–11 months).

The characteristics of the included patients are summarized in Table 1. The majority were male (78.7%), with females accounting for 21.3%. Most patients (76.6%) were under the age of 70, with a mean age of 62 years. A total of 88% of the patients had an ECOG performance status of 1 or 2, reflecting relatively good functional status. In terms of cancer type, oral cavity cancer was the most frequent, comprising 52.4% of cases. Of the 286 patients, 134 (46.9%) had locally advanced disease, while 152 (53.1%) presented with metastatic disease. Among the metastatic cases, 47 patients (30.9%) had bone metastases.

**TABLE 1:** Characteristics of patients.

Variable name	Level	Overall	Hypercalcemia	Normocalcemia	*p*
*n*		286	51 (17.8)	235 (82.2)	
Sex	Female	61 (21.3)	14 (27.5)	47 (20.0)	0.323
	Male	225 (78.7)	37 (72.5)	188 (80.0)	
Age category	< 50	40 (13.7)	8 (15.7)	32 (13.6)	0.224
	50–70	179 (62.6)	17 (33.3)	68 (28.9)	
	> 70	67 (23.4)	10 (19.6)	84 (35.7)	
ECOG	0	14 (4.9)	1 (2.0)	13 (5.6)	0.372
	1	143 (50.5)	23 (45.1)	120 (51.7)	
	2	106 (37.1)	21 (41.2)	85 (36.6)	
	> 2	20 (7.1)	6 (11.8)	14 (6.0)	
Tumor site	Hypopharynx	35 (12.2)	6 (11.8)	29 (12.3)	0.155
	Larynx	35 (12.2)	6 (11.8)	29 (12.3)	
	Oral cavity	150 (52.4)	33 (64.7)	117 (49.8)	
	Oropharynx	66 (23.1)	6 (11.8)	60 (25.5)	
CPS category	< 1	14 (18.4)	2 (13.3)	12 (19.7)	0.728
	1–19	34 (44.7)	8 (53.3)	26 (42.6)	
	≥ 20	28 (36.8)	5 (33.3)	23 (37.7)	
Metastatic status	Locally advanced	134 (46.9)	30 (58.8)	104 (44.3)	0.083
	Metastatic	152 (53.1)	21 (41.2)	131 (55.7)	
Bone metastasis	No	239 (83.6)	42 (82.4)	197 (83.8)	0.960
	Yes	47 (16.4)	9 (17.6)	38 (16.2)	
Osteolysis at diagnosis	No	132 (67.7)	17 (45.9)	115 (72.8)	0.003
	Yes	63 (32.3)	20 (54.1)	43 (27.2)	
Calcemia		2.4 (0.3)	2.9 (0.4)	2.3 (0.1)	< 0.001
Calcemia category	Normocalcemia	235 (82.2)	0 (0.0)	235 (100.0)	< 0.001
	Hypercalcemia	43 (15.0)	43 (84.3)	0 (0.0)	
	Hypocalcemia	8 (2.8)	8 (15.7)	0 (0.0)	
First line of treatment	CT‐ICI	29 (10.1)	7 (13.7)	22 (9.4)	0.320
	Extreme	173 (60.5)	29 (56.9)	144 (61.3)	
	ICI	49 (17.1)	10 (19.6)	39 (16.6)	
	TPEx	21 (7.3)	5 (9.8)	16 (6.8)	
	Other	14 (4.9)	0 (0.0)	14 (6.0)	
Treatment discontinuation	No	263 (92.0)	38 (74.5)	225 (95.7)	< 0.001
	Yes	23 (8.0)	13 (25.5)	10 (4.3)	
Treatment dose reduction	Cardiac	3 (4.9)	1 (5.9)	1 (2.3)	0.005
	Digestive	4 (6.5)	1 (5.9)	0 (0.0)	
	General detei	12 (19.7)	2 (11.8)	8 (18.6)	
	Hemorrhage	12 (19.7)	7 (41.2)	5 (11.4)	
	Hematological	29 (47.5)	2 (11.8)	19 (61.4)	
	Hepatic	1 (1.6)	0 (0.0)	1 (2.3)	
	Infection	4 (6.6)	3 (17.6)	1 (2.3)	
	Skin	7 (11.5)	0 (0.0)	5 (11.4)	
Number of treatment line	1	144 (50.3)	26 (51.0)	118 (50.2)	0.514
	≥ 2	141 (49.4)	24 (47.0)	123 (49.7)	

### 3.2 Incidence of Hypercalcemia and Associated Clinical Features

At diagnosis, hypercalcemia was identified in 51 out of 286 patients (17.8%). Most cases were of grade 1 or 2 severity. The presence of hypercalcemia was not significantly associated with the disease stage or the presence of bone metastases. No differences were observed in performance status or tumor localization between hypercalcemic and normocalcemic patients. Hypercalcemia was significantly associated with local osteolysis, as 20 patients (51.4%) in the hypercalcemic group exhibited local osteolysis compared to 43 patients (27.2%) in the non‐hypercalcemic group (*p* = 0.003). Treatment discontinuation, defined as the cessation of systemic therapy prior to completing the planned regimen, was also more frequent, with 13 hypercalcemic patients (25.5%) who failed treatment completion versus 10 (4.3%) in the non‐hypercalcemic group (*p* < 0.001).

### 3.3 Association Between Hypercalcemia and Overall Survival

**FIGURE 2:**
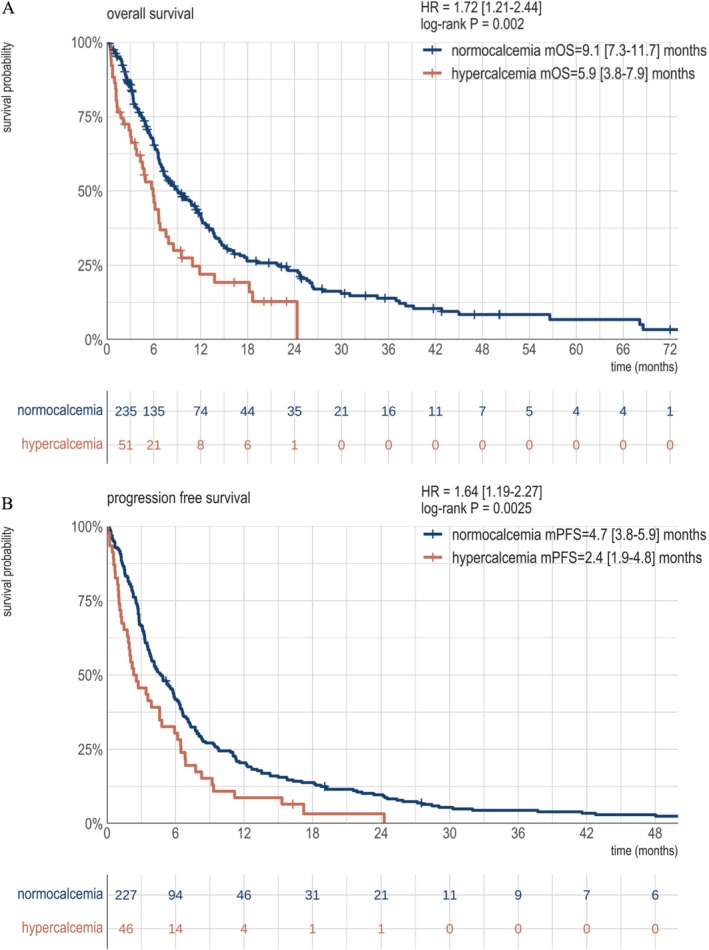
Kaplan–Meier survival curves for overall survival (panel A) and progression‐free survival (panel B) depending on calcemic status. [Color figure can be viewed at wileyonlinelibrary.com]

**FIGURE 3:**
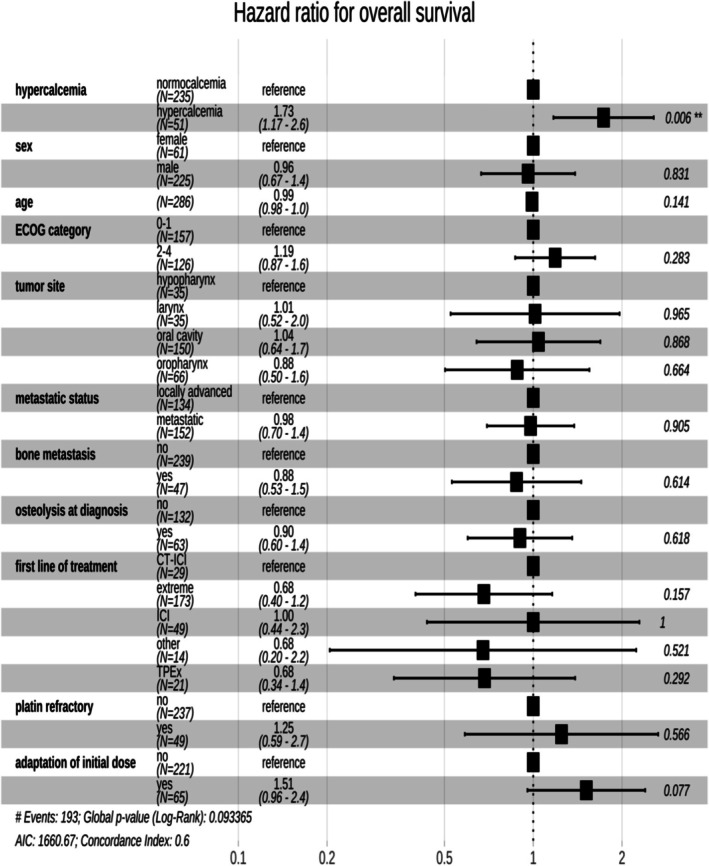
Multivariate analysis of overall survival.

The median overall survival (OS) for the entire cohort was 7.9 months (95% CI = 6.8–10.6). Hypercalcemia at diagnosis was associated with significantly shorter OS. Patients with hypercalcemia had a median OS of 5.9 months (95% CI = 3.8–7.9 months), compared to 9.1 months (95% CI = 7.3–11.7 months) for those without hypercalcemia (*p* = 0.002, Figure 2A). In multivariate analysis, after adjusting for clinical, tumoral, and therapeutic factors, hypercalcemia remained an independent prognostic factor, with a hazard ratio (HR) of 1.73 (95% CI = 1.17–2.56), (*p* = 0.006 Figure 3).

### 3.4 Association Between Hypercalcemia and Progression‐Free Survival

**FIGURE 4:**
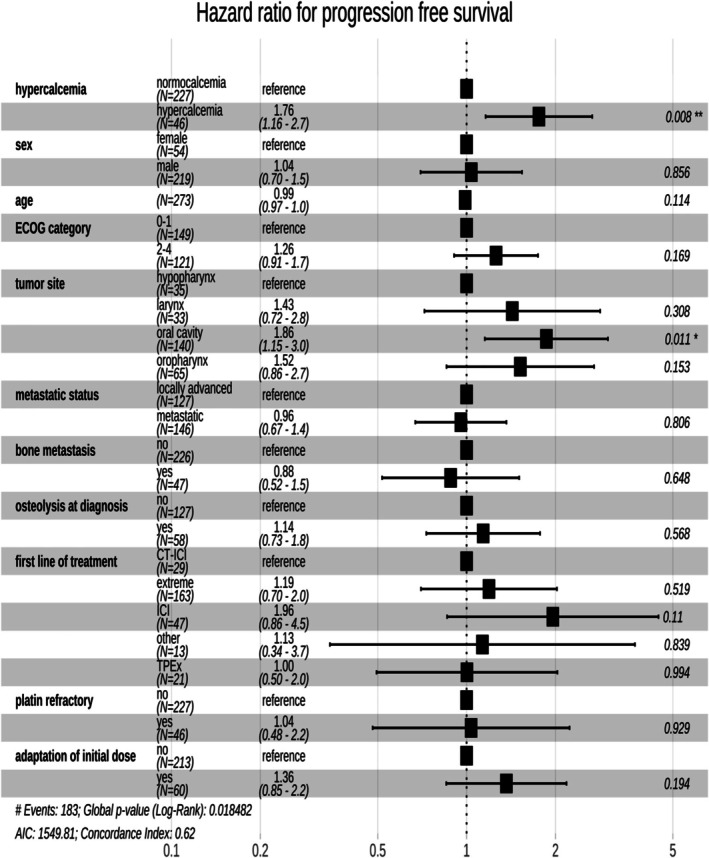
Multivariate analysis of progression‐free survival.

The median progression‐free survival (PFS) for the cohort was 4.3 months [3.6–5.5 months]. Hypercalcemia was associated with significantly shorter PFS, with a median PFS of 2.4 months (95% CI = 1.9–4.8 months) with hypercalcemia, compared to 4.7 months (95% CI = 3.8–5.9 months) without hypercalcemia (HR = 1.64, *p* = 0.0025, Figure 2B). In multivariate analysis, after adjusting for other factors, hypercalcemia continued to have a significant detrimental effect on PFS, with a hazard ratio (HR = 1.76, 95% CI = 1.16–2.7, *p* = 0.008). There was no significant independent effect of ECOG status, metastatic status, or first‐line treatment on PFS. However, the presence of an oral cavity primary tumor was associated with significantly shorter PFS (HR = 1.86 [1.15–3.0], *p* = 0.011, Figure 4).

### 3.5 Management of Hypercalcemia

Among the hypercalcemic patients, parathyroid hormone (PTH) levels were measured in 20 patients (38.4%), with seven cases showing non‐measurable PTH levels. PTH‐related protein (PTH‐rP) levels were assessed in seven patients (13.7%), with three showing elevated levels. None of these measurements were performed in normocalcemic patients.

Regarding treatment, 19 hypercalcemic patients (38%) received bisphosphonates, while 32 patients (62.2%) were treated with hydration alone.

## 4 Discussion

In the present study, we accrued a retrospective cohort of patients diagnosed with a HNSCC locally advanced or metastatic to evaluate hypercalcemia at diagnosis and its link with patient outcomes. In this real‐world study, we found an incidence of hypercalcemia of 17.8% and that hypercalcemia was independently correlated with poorer survival and treatment efficacy.

First, we found a similar hypercalcemia rate to a previous study from Penel et al., who reported a 23.5% incidence of hypercalcemia in head and neck cancer patients at the onset of treatment [12]. As in our study, the incidence of bone metastases was relatively low, with 20 out of 136 patients (14.5%) affected. Additionally, 32 cases of hypercalcemia were observed at the time of cancer diagnosis. Similarly, Alsifary et al. found hypercalcemia in 51% of patients with HNSCC over a 4‐year period [11], highlighting that hypercalcemia is a relatively common complication in this patient population. The median survival from the first hypercalcemic episode was 74 days (95% CI, 0–234). This relatively short survival may reflect the fact that patients presenting with hypercalcemia often have more advanced or severe disease at an earlier stage, independently of their calcium levels. Moreover, the treatments available during the study period were likely less effective compared to current therapeutic options. This is supported by the observation that the overall survival of non‐hypercalcemic patients in this study was only 128 days, further indicating a generally worse prognosis for this cohort. Our analysis focused specifically on hypercalcemia present at diagnosis to assess its impact at the initiation of systemic therapy. The majority of patients were male (78.7%), which is consistent with the epidemiology of HNSCC, and oral cavity cancers accounted for 52.4% of cases, likely due to the recruitment bias from the maxillofacial surgery department. The cohort represented a general HNSCC population, with 88% having a favorable ECOG performance status (0–2). There was no significant difference between the hypercalcemia and normocalcemia groups in terms of baseline characteristics, including ECOG status, disease stage, or metastatic spread.

Second, we found that hypercalcemia was associated with local osteolysis but not bone metastasis, suggesting a potential role of local bone destruction in the pathogenesis of hypercalcemia. This observation raises the possibility that local osteolysis may contribute to the release of calcium from bone, exacerbating hypercalcemia, while in other cases, it may be due to paraneoplastic syndrome. Further investigation into the association between paraneoplastic syndromes and local osteolysis could provide insights into the biological mechanisms underlying hypercalcemia in HNSCC [13]. Tumor‐derived factors, such as parathyroid hormone‐related protein (PTH‐rP), are known contributors to hypercalcemia in malignancies [14, 15, 16], and their role in this context warrants further exploration.

**FIGURE 5:**
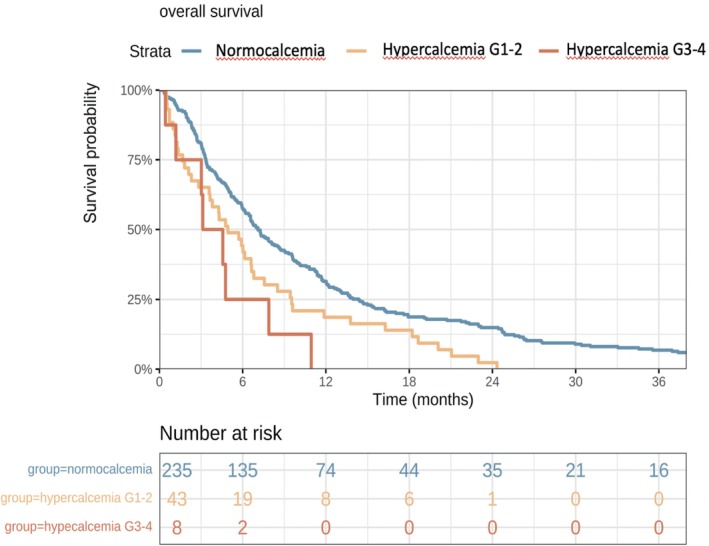
Kaplan–Meier curve for overall survival stratified by hypercalcemia grade. Hypercalcemia grade was stratified according to CTCAE 5.0 (common terminology criteria for adverse events). [Color figure can be viewed at wileyonlinelibrary.com]

Third, the presence of hypercalcemia was strongly associated with worse overall survival (OS) and progression‐free survival (PFS). It was the only significant prognostic factor identified in this study (Figure 3). Although the impact on survival appeared to be more pronounced for patients with grade 3 or 4 hypercalcemia, the small sample size limits the interpretation of these findings (Figure 5). Hypercalcemic patients had a significantly shorter OS, and this association remained significant after adjusting for confounding clinical and initial therapeutic factors. Hypercalcemia was identified as the sole independent prognostic factor in our cohort, consistent with previous studies [17]. In comparison, the overall survival in our cohort (7.8 months) was shorter than that reported in clinical trials, such as Burtness et al., who reported a median OS ranging from 10.9 to 14.3 months [18]. This discrepancy could be attributed to the fact that our study reflects a real‐world population, which may differ from the highly selected populations typically enrolled in therapeutic trials.

Hypercalcemia was also significantly associated with a shorter PFS, with hypercalcemic patients showing a median PFS of 2.4 months compared to 4.7 months in normocalcemic patients, and this association persisted in multivariate analysis. Additionally, primary tumors located in the oral cavity were associated with shorter PFS, possibly due to the local–regional progression often seen in these cancers, which is frequently accompanied by local osteolysis, infection, and hemorrhage, all of which can negatively impact disease control and survival. The presence of hypercalcemia did not influence the choice of the oncological treatment proposed, combined chemotherapy and immunotherapy (13.7% vs. 9.4% for hypercalcemia group vs. normocalcemia group respectively, *p* = 0.31), EXTREME regimen (56.9% vs. 61.3%, *p* = 0.63) or ICI alone (19.6% vs. 16.6%, *p* = 0.68). Furthermore, hypercalcemia was significantly associated with early treatment discontinuation; 26.9% of hypercalcemic patients prematurely halted treatment compared to 4.3% of normocalcemic patients (*p* < 0.001). This early discontinuation was frequently due to a decline in the general condition, observed in 47.1% of hypercalcemic patients (*p* < 0.01). Several hypotheses could explain this. Hypercalcemia could reflect tumor intrinsic aggressiveness, which explains the link with osteolysis. The rapid deterioration could also be explained by hypercalcemia‐induced anorexia, although there was no significant difference in ECOG performance status between the two groups, and ECOG did not emerge as a prognostic factor in our study.

The main limitation of our study lies in its retrospective nature. Therefore, in 60.8% of cases, an etiological workup was not performed. In our study, PTH levels were measured for 20 patients and were lowered by negative feedback when measured (between 6 and First ng/L). Although ectopic hyperparathyroidism is rare in cancer‐associated hypercalcemia, the possibility of concurrent primary hyperparathyroidism cannot be excluded. A prior study reported that 6% of cancer patients with hypercalcemia had underlying primary hyperparathyroidism [19]. Moreover, PTH‐rP, which is responsible for hypercalcemia in approximately 50% of cancer cases [14, 20, 21], was infrequently measured. Vitamin D levels, which could exacerbate hypercalcemia through feedback mechanisms with PTH, were assessed in only 43.1% of cases. These gaps in diagnostic testing highlight the need for a more standardized approach to evaluating hypercalcemia in cancer patients. Additionally, given the relatively small sample size of hypercalcemic and hypocalcemic patients, potential inaccuracies in multivariate modeling should be considered when adjusting for multiple covariates.

Currently, no professional guidelines specifically address the management of hypercalcemia in cancer patients. In practice, treatment is generally based on three principles: Correcting dehydration, inhibiting bone resorption, and addressing the underlying malignancy [22]. In our cohort, all hypercalcemic patients received hydration therapy, while 38.2% were treated with bisphosphonates. Notably, bisphosphonate use was associated with lower survival, which may reflect its use in patients with more severe hypercalcemia (≥ grade 3). A similar finding was reported in a previous retrospective study [12]. Emerging evidence suggests that vitamin D supplementation could also play a role in the management of hypercalcemia to avoid hypocalcemia, which may be induced by bisphosphonates, but this requires further study [23]. The risk of osteoradionecrosis, particularly in patients with a history of head and neck radiotherapy, further complicates the decision to use anti‐resorptive therapies like bisphosphonates.

In conclusion, our study highlights the significant prognostic value of hypercalcemia in HNSCC patients, demonstrating its association with both reduced OS and PFS. Standardizing diagnostic evaluations and therapeutic interventions for hypercalcemia could improve patient outcomes and should be prioritized in future clinical guidelines.

## Ethics Statement

This retrospective observational study complies with the French regulation MR004 for which no ethical approval is required. The database of the study was declared to the “Commission Nationale de l'Informatique et des Libertés” (CNIL N° CERFA 13810*01).

## Data Availability

The data supporting the findings of this study will be made available in a public repository (Pubmed) after the acceptance and publication of this article. A DOI and reference number will be assigned at that time.
